# Multimodal identification of a rare head and neck cancer patient cohort in the clinical data warehouse of Greater Paris Teaching Hospital

**DOI:** 10.1016/j.esmorw.2025.100151

**Published:** 2025-05-29

**Authors:** A. La Rosa, M. Verdoux, P. Riebler, I. Lolli, C. Daniel, X. Tannier, S. Atallah, B. Baujat, E. Kempf

**Affiliations:** 1 Sorbonne University, Inserm, Université Sorbonne Paris-Nord, LIMICS, INSERM U 1142, Paris, France; 2 Otolaryngology-Head and Neck Surgery Department, Hospital Tenon, AP-HP, Sorbonne University, Paris, France; 3 Clinical Research Unit, Direction of Clinical Research, Bicêtre Hospital, AP-HP, Paris-Saclay University, Le Kremlin-Bicêtre, France; 4 Département d’Oncologie Médicale, Assistance Publique—Hôpitaux de Paris, UPEC, GHU Henri Mondor, Créteil, France

**Keywords:** head and neck cancer, rare cancer, natural language processing, clinical data warehouse

## Abstract

**Background:**

Ten percent of head and neck cancers (HNCs) differ from the common upper aerodigestive tract squamous-cell carcinoma. These rare HNCs can be rare because of their histology or anatomical location. The federation of clinical data warehouses (CDWs) holds potential for advancing our understanding of these pathologies. This study aimed to develop a multimodal algorithm to identify rare HNC patients in a CDW.

**Materials and methods:**

We carried out a cross-sectional study on the CDW of a conglomerate of 38 university hospitals. We developed a multimodal classification algorithm to identify rare HNC patients by integrating International Classification of Diseases, 10th revision (ICD-10) codes, Association for the Development of Computer Science in Cytology and Pathological Anatomy (ADICAP) codes and free-text data from pathology reports using natural language processing (NLP). Algorithm performance was evaluated by an HNC medical expert using a validation set of 100 manually annotated cases.

**Results:**

Of 333 852 cancer patients, 9141 were identified as HNC patients based on ICD-10 and ADICAP codes. The multimodal algorithm using ICD-10 or ADICAP codes or NLP-processed free text classified 4515 patients as rare HNC patients, with 2168 identified by a minimum of two data sources. It showed a 91% sensitivity and a 95% specificity when relying on multiple data sources, with a 76% positive predictive value observed for rare histology identification compared with 43% for rare topography.

**Conclusions:**

This study demonstrates the feasibility and utility of a multimodal electronic health record-based approach to identify rare HNC patients in a CDW. Incorporating free-text and structured data improves the reliability of such cohort identification.

## Highlights

• Three data sources were used to identify rare HNC patients in a CDW.
• A total of 4515 cancer patients were classified as having rare HNC via ICD-10, ADICAP or NLP.
• A subset of 2168 patients was identified by at least two data sources.
• When relying on multiple data sources, 91% sensitivity and 95% specificity were reached.
• NLP performed well in terms of sensitivity, but showed a 9% rate of false positives.

## Introduction

Most head and neck cancers (HNCs) are squamous-cell carcinomas (SCCs) of the upper aerodigestive tract, for which the main risk factors are smoking, alcohol consumption and human papillomavirus infection in the oropharynx. Rare HNCs are rare in terms of histology and/or location and account for <5 people in 10 000 and <10% of HNCs, corresponding to ∼49 000 patients per year in Europe.^1^^,^^2^ The natural history and prognosis of these rare HNCs vary considerably from SCCs. Moreover, the rarity of these cancers is associated with a lack of guidelines in routine care. Due to the predominance of small, heterogeneous and potentially biased studies, and the rarity of randomized clinical trials, the therapeutic strategy is based on a low level of medical and scientific evidence, justifying the constitution of large cohorts of rare HNC patients. Against this backdrop, medical and scientific teams have joined forces at a European level to improve epidemiological, biological and clinical knowledge and the therapeutic management of these diseases, through the creation of registries and research projects such as the European Reference Network for rare adult solid cancers (EURACAN) registry of rare HNCs.^3^

The development of hospital clinical data warehouses (CDWs) containing patient electronic health records (EHRs) presents an opportunity to go further. Structured data such as International Classification of Diseases, 10th revision (ICD-10) codes and claims data can be used but have shown their low sensitivity (Se) for identifying cancer disease status, with Se between 50% and 60%.^4^, ^5^, ^6^ Beyond structured data, most of the information in the EHR is contained in the free text of the patient EHR, such as the HNC pathology type. The exploitation of the EHR on a large scale therefore presupposes the ability to automatically extract data from free text, using natural language processing (NLP) algorithms. Combining free-text data and structured data could be a perspective for targeting patients with rare diseases such as rare HNC.

The objective of this study was to identify a cohort of rare HNC patients, based on multimodal information retrieval algorithms using hospital EHR data.

## Materials and methods

### Design and ethics

We conducted a multicentre cross-sectional study on the hospital EHR data available in the Assistance Publique–Hôpitaux de Paris (AP-HP) CDW. The AP-HP CDW contains the data of 11.4 million patients, collected during their care in all 38 AP-HP university hospitals. The constitution of the CDW of the AP-HP was authorized by the Commission Nationale de l’Informatique et des Libertés (CNIL) on 19 January 2017 (authorization no. 19800120). The research work presented here was approved by the Scientific and Ethical Committee of AP-HP (IRB 00011591, 5 September 2022) on 15 May 2020 (authorization CSE-EDS no. 22-24_OncOMOP Terabase). We reported the study results according to the RECORD statement (Supplementary Figure S1, available at https://doi.org/10.1016/j.esmorw.2025.100151).

### Data sources

In this study, we used claims data from the Programme de Médicalisation des Systèmes d’Information (PMSI, the national hospital claims database) and clinical data contained in the patient EHRs [pathology reports and multidisciplinary meeting (MDM) reports]. The PMSI contains structured data including the coding of diagnoses according to ICD-10. ICD-10 codes were directly computable from the database. Each pathology report contains a normalized code related to the French Association pour le Développement de l’Informatique en Cytologie et en Anatomie Pathologique (ADICAP, Association for the Development of Computer Science in Cytology and Pathological Anatomy). Those codes are made of eight characters, among which two are dedicated to the description of the sample site (e.g. sinus), two are dedicated to the benign versus malignant nature of the sample, two are dedicated to the tumour histology type and two are dedicated to the type of sample (e.g. biopsy). We extracted the ADICAP codes from pathology reports using NLP algorithms available in the AP-HP CDW libraries.^7^

Through the collaboration of an HNC medical expert (ALR) and a senior data scientist (MV), we developed regular expressions to retrieve free-text key data (histological type of cancer and its topography) using 600 medical reports (pathology reports and MDM reports) (development set) and validated the approach using an additional 200 medical reports. Those medical reports were randomly selected from the EHRs of HNC patients. Validation was carried out manually by an HNC medical expert based on pathology reports and MDM reports. The developed NLP algorithms used components from the EDS-NLP v0.7.4 software library, handling hypotheses, medical history and negation text constraints.^8^

### Source population and constitution of an HNC patient cohort

We included a prevalent cohort of patients among AP-HP CDW with cancer-related hospitalizations based on ICD-10 codes (Supplementary Table S1, available at https://doi.org/10.1016/j.esmorw.2025.100151). Extraction was carried out on 12 March 2024. Data were available in an Observational Medical Outcomes Partnership—Common Data Model (OMOP-CDM) v5.3 format.

We identified patients with an ICD-10 code (as a primary diagnosis or related diagnosis) or ADICAP code related to HNC (Supplementary Table S2, available at https://doi.org/10.1016/j.esmorw.2025.100151). We calculated the number (%) of patients for whom at least one hospital pathology report was available. The HNC patient cohort was defined as patients with at least one hospital pathology record available and an HNC ICD-10 code or ADICAP code.

### Extraction of rare HNC topography and histology variables from the patient hospital pathology reports

According to the French network of expertise in rare HNCs (Réseau d’Expertise Français des Cancers ORL Rares, REFCOR network), rare HNC cases were defined by the occurrence of a so-called rare topography (salivary glands, sinus, nasal fossa, nasopharynx, middle ear) and/or a rare histology (any type excluding SCC). Among the HNC patient cohort, we extracted the following two variables from each hospital pathology report: rare topography HNC case (yes/no) and rare histology HNC case (yes/no).

We extracted the variable ‘rare HNC topography’ from each hospital pathology report using free text and ADICAP-based regular expressions (Supplementary Tables S3 and S4, available at https://doi.org/10.1016/j.esmorw.2025.100151).

We extracted the variable ‘rare HNC histology’ from hospital pathology reports:

- Free text mentioning a rare tumour histology (excluding SCC) in any HNC topography,
- ADICAP normalized codes related to malignant tumours in any HNC topography with a rare histology (Supplementary Tables S3 and S4, available at https://doi.org/10.1016/j.esmorw.2025.100151).

For each extracted variable, the data source was identified (free text and/or ADICAP).

### Building a multimodal algorithm to identify a cohort of rare HNC patients

We built the rare HNC patient cohort as the union of the two following subcohorts:

#### ‘Rare topography HNC’ patient subcohort

Each patient with at least one extracted ‘rare HNC topography’ variable from a pathology report regardless of the data source (ADICAP code and/or free text) or one ICD-10 code related to rare topography HNC diagnoses (C07, C08, C11, C41, C300, C301, C31) in primary or related or associated diagnosis in the PMSI was included in the ‘rare topography HNC’ patient subcohort.

#### ‘Rare histology HNC’ patient subcohort

Each patient with at least one extracted ‘rare HNC histology’ variable from a pathology report regardless of the data source (ADICAP code and/or free text) was included in the ‘rare histology HNC’ patient subcohort.

The data sources (free text and/or ADICAP and/or ICD-10) were reported for each patient.

We defined the ‘consolidated rare HNC patient cohort’ based on the concordance of at least two data sources (ADICAP code, ICD-10 code, free text). The method is summarized in Supplementary Figure S2, available at https://doi.org/10.1016/j.esmorw.2025.100151.

### Assessment of the performances of the multimodal algorithm

An HNC medical expert (PR) annotated and classified the EHRs of 100 HNC patients that were not already used during the iteration process, according to the presence of a rare HNC or not, using the ICD-10 code and the pathology report data (ADICAP code and free text) only. We measured Se, specificity (Sp) and positive and negative predictive values (PPV and NPV) of the multimodal algorithm for identifying the rare HNC patient cohort, the consolidated rare HNC patient cohort and the rare topography and rare histology HNC patient subcohorts. The external evaluation was also carried out on each data source.

## Results

### Source population and constitution of an HNC patient cohort

**Figure 1:**
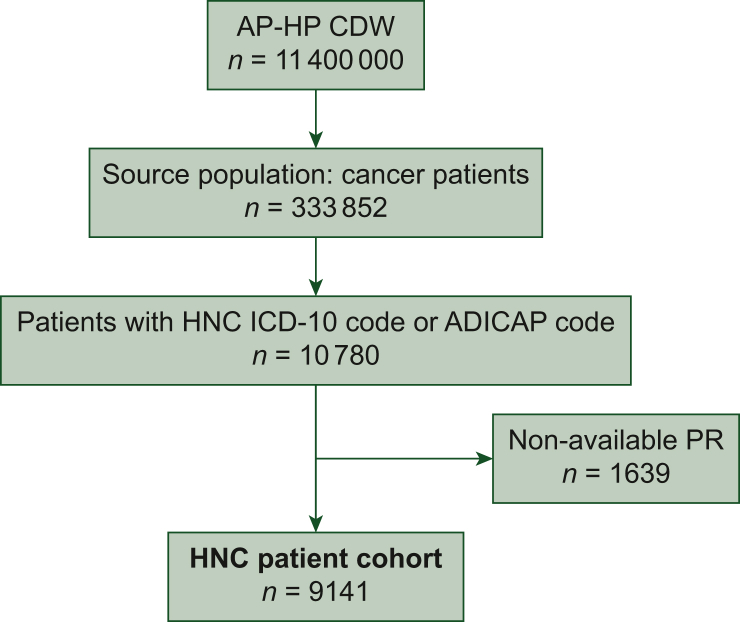
**Flow chart.** ADICAP, Association pour le Développement de l’Informatique en Cytologie et en Anatomie Pathologique, Association for the Development of Computer Science in Cytology and Pathological Anatomy; AP-HP CDW, Greater Paris Teaching Hospital (Assistance Publique–Hôpitaux de Paris, AP-HP) clinical data warehouse; HNC, head and neck cancer; ICD-10, International Classification of Diseases, 10th revision; PR, pathology report.

Of 333 852 cancer inpatients referred to AP-HP from 1990 to 2024, 10 780 had an ICD-10 code or an ADICAP code related to an HNC. Among them, 9141 (85%) had an available hospital pathology report and constituted the HNC patient cohort (Figure 1). The cohort patients had the following characteristics: the median age was 64 years [interquartile range (IQR) 55-73 years], and the sex ratio (male : female) was 2 : 1.

Extraction of rare HNC topography and histology variables from the patient hospital pathology reports and building of a multimodal algorithm to identify a cohort of rare HNC patients

**Figure 2:**
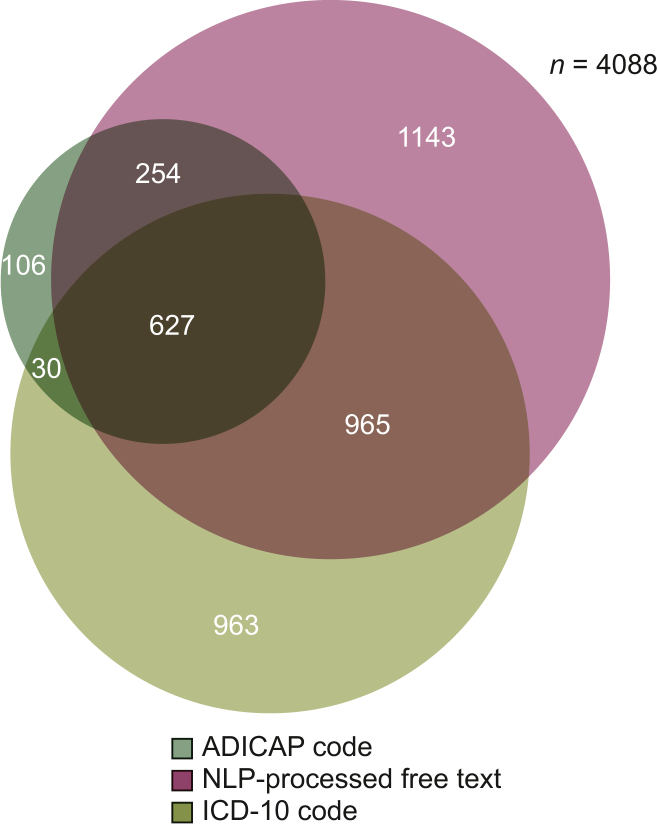
**Rare topography HNC patient subcohort: repartition by data source.** ADICAP, Association pour le Développement de l’Informatique en Cytologie et en Anatomie Pathologique, Association for the Development of Computer Science in Cytology and Pathological Anatomy; HNC, head and neck cancer; ICD-10, International Classification of Diseases, 10th revision; NLP, natural language processing.

**Figure 3:**
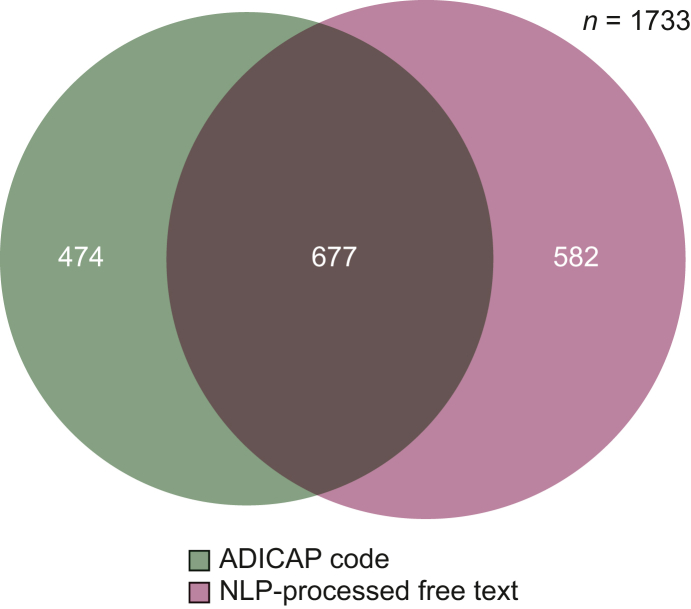
**Rare histology HNC patient subcohort: repartition by data source.** ADICAP, Association pour le Développement de l’Informatique en Cytologie et en Anatomie Pathologique, Association for the Development of Computer Science in Cytology and Pathological Anatomy; HNC, head and neck cancer; NLP, natural language processing.

**Figure 4:**
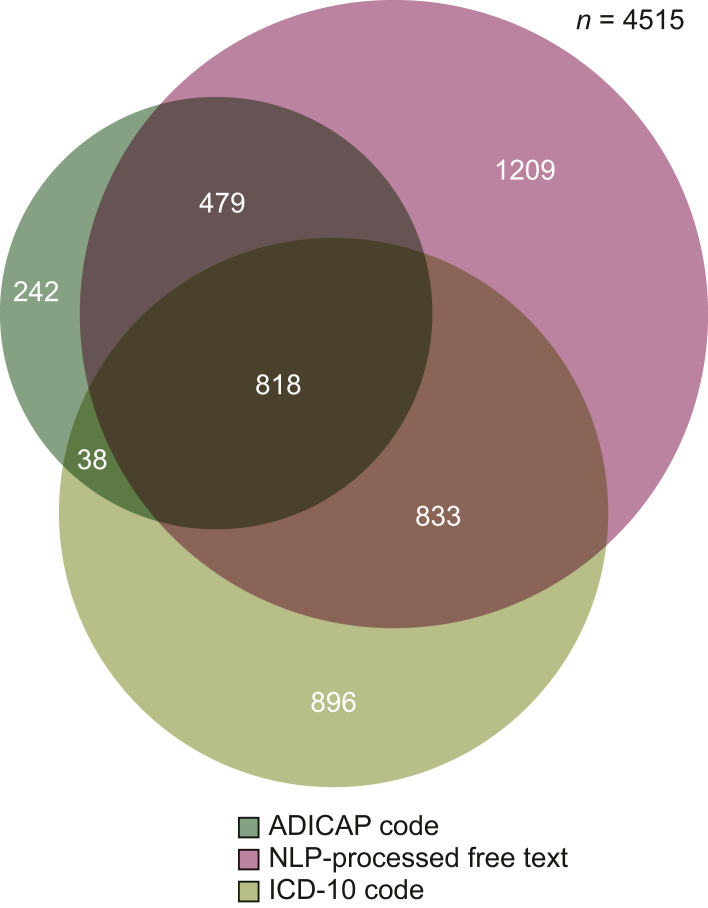
**Rare HNC patient cohort: repartition by data source.** ADICAP, Association pour le Développement de l’Informatique en Cytologie et en Anatomie Pathologique, Association for the Development of Computer Science in Cytology and Pathological Anatomy; HNC, head and neck cancer; ICD-10, International Classification of Diseases, 10th revision; NLP, natural language processing.

The ‘rare topography HNC’ patient subcohort included 4088 patients. Repartition of identification data source is represented in Figure 2. The ‘rare histology HNC’ patient subcohort included 1733 patients. Repartition of identification data source is represented in Figure 3. Pooling these two subcohorts, 4515 patients constituted the rare HNC patient cohort with a median age of 62 years (IQR 51-72 years) and a sex ratio of 2 : 2. Repartition of identification data source is represented in Figure 4. We identified 2168 patients by at least two data sources as the ‘consolidated rare HNC patient cohort’ (Figure 2; Supplementary Figure S3, available at https://doi.org/10.1016/j.esmorw.2025.100151). All patients but 38 of the consolidated rare HNC patient cohort were identified by NLP at least, but associating at least one other data source allowed a better Sp than relying on NLP code only (Sp 95% versus 88%). Supplementary Table S5, available at https://doi.org/10.1016/j.esmorw.2025.100151, breaks down the analysis of the pertinence of each data source in each subcohort and confirms that adding ADICAP code (and ICD-10 code for the rare topography HNC patient subcohort) is beneficial in terms of Sp and PPV.

### Assessment of the performances of the multimodal algorithm

The performances of the multimodal algorithm developed to constitute the rare HNC patient cohort, its subcohorts, and the consolidated rare HNC patient cohort depending on data sources (ICD-10 code, ADICAP, free text) and for each subcohort are displayed in Tables 1 and 2.

**Table 1:** Performance metrics of the algorithm for identifying rare HNC patient cohort depending on data sources

Performance by data source	TP	FP	FN	TN	Total	Sensitivity (%)	Specificity (%)	PPV (%)	NPV (%)
ICD-10 code	15	9	7	69	100	68	88	62	91
ADICAP code	14	3	8	75	100	64	96	82	90
NLP-processed free text	21	9	1	69	100	95	88	70	99

ADICAP, Association pour le Développement de l’Informatique en Cytologie et en Anatomie Pathologique, Association for the Development of Computer Science in Cytology and Pathological Anatomy; FN, false negative; FP, false positive; HNC, head and neck cancer; ICD-10, International Classification of Diseases, 10th revision; NLP, natural language processing; NPV, negative predictive value; PPV, positive predictive value; TN, true negative; TP, true positive.

**Table 2:** Performance metrics of the algorithm for identifying rare HNC patient cohort, consolidated rare HNC patient cohort and rare topography and rare histology HNC patient subcohorts

Performance by cohort or subcohort	TP	FP	FN	TN	Total	Sensitivity (%)	Specificity (%)	PPV (%)	NPV (%)
Rare HNC patient cohort	22	25	0	53	100	100	68	47	100
Rare topography HNC patient subcohort	18	24	0	58	100	100	71	43	100
Rare histology HNC patient subcohort	16	5	2	77	100	89	94	76	97
Consolidated rare HNC patient cohort	20	4	2	74	100	91	95	83	97

Consolidated rare HNC patient cohort corresponds to the patients identified with a rare HNC on at least two data sources among ADICAP, ICD-10 and NLP-processed free text.

ADICAP, Association pour le Développement de l’Informatique en Cytologie et en Anatomie Pathologique, Association for the Development of Computer Science in Cytology and Pathological Anatomy; FN, false negative; FP, false positive; HNC, head and neck cancer; ICD-10, International Classification of Diseases, 10th revision; NLP, natural language processing; NPV, negative predictive value; PPV, positive predictive value; TN, true negative; TP, true positive.

The Sp, Se and NPV of the rare HNC patient cohort algorithm were 68%, 100% and 100%, respectively. PPV for detecting rare histology was 76% compared with 43% for rare topography. Free-text and ADICAP data sources were associated with a PPV of 70% and 82%, respectively, whereas ICD-10 code identification PPV reached 62%. As for the algorithm for constituting the consolidated rare HNC patient cohort, the Sp reached 95%, versus 68% for the rare HNC patient cohort.

## Discussion

In this study, which is, to our knowledge, the first of its kind, we developed and evaluated a multimodal algorithm using EHR data to identify patients with rare HNCs. Our findings highlight both the potential and limitations of using hospital CDWs for cohort identification, especially in rare cancer populations. One of the key strengths of this approach lies in its ability to exploit multimodal data sources—ICD-10 codes, ADICAP codes and free-text pathology reports—allowing for more accurate patient identification. Our results showed that the algorithm developed to constitute a so-called ‘consolidated’ rare HNC patient cohort achieved an Se of 91% and an Sp of 95% when relying on at least two data sources. This is a significant improvement compared with single-source reliance, such as using ICD-10 codes alone, which had a notably lower performance (Se 68%, Sp 88%). These findings are consistent with prior research suggesting that incorporating free-text data and structured codes enhances the accuracy of EHR-based algorithms.^9^, ^10^, ^11^

However, there are several issues to consider. One concern raised is the unexpectedly high proportion of patients classified as rare HNC in our study. Indeed, 49% (4515) of the 9141 HNC patients were initially classified as rare HNC, and 24% (2168) under the definition of the ‘consolidated rare HNC patient cohort’, which is much higher than the 10% of HNCs typically classified as rare. It is notable that it was also the case in the validation sample. This could be caused by several biases. Firstly, the AP-HP CDW includes hospitals labelled as reference centres for rare cancers. Secondly, some cancer topographies are subject to debate in terms of classification. For example, a cancer invading both the oral cavity and the maxillary sinus could be classified as a sinus cancer, hence a rare cancer or a common oral cavity cancer, and is likely to be subject to difference in classification among experts. This hypothesis fits with the high number of rare topography HNC patients identified among the cohort.

The algorithm’s performance may also vary depending on the quality and completeness of the underlying EHR data, as missing data are a common issue in CDWs. The availability of data sources relies on the complex infrastructure of the AP-HP CDW, which can generate substantial loss of key information.^12^

Each data source has its limitations. More specifically, ICD-10 code is only available for inpatient stays, does not inform on histology and is prone to coding mistakes due to contiguous topography or metastasis. It is hence important to note that ICD-10 code identification is inherently limited by its ability to provide topography data without any histological data, artificially reducing its Se in our analysis. Supplementary Table S5, available at https://doi.org/10.1016/j.esmorw.2025.100151, shows the performances of ICD-10 code in the rare topography HNC patient cohort identification alone with better Se (78%) and NPV (95%). The use of ADICAP codes or of NLP-processed free text depends on the pathology report availability, which was 85% in our source population. It also only provides information on the biopsy site and not on the primary tumour site. As for free text managed by NLP, false positives are frequent due to mistakes in identification of negation, history or hypothesis.^13^ In our analysis, false-positive rate using the NLP identification was 9%. However, it is a very sensitive method with only 1% rate of false negatives and a good NPV. In each subcohort, its performances seem to be quite similar, as shown in Supplementary Table S5, available at https://doi.org/10.1016/j.esmorw.2025.100151. This observation does not encourage us to privilege the exclusive use of this method in one subcohort or the other, but data on a larger population could lead to different conclusions.

Regarding data extracted from free-text documents by NLP, the availability of data depends on the availability of the relevant text files, the presence of the information of interest and the performance of the algorithm in extracting these data.^14^^,^^15^ It is interesting to note that our algorithm demonstrated better precision for rare histology identification compared with rare topography, possibly due to the less standardized medical reporting practices for anatomical location.

Hence, solutions could be to improve availability of pathology reports and their completeness through data quality campaigns; to multiply the sources; and to improve the algorithms used. Vigilance must be also maintained to allow longevity of algorithms.^16^

The performance of the algorithms would probably be improved by increasing the size of the annotated set for development and validation. However, this annotation process is time consuming and requires significant investment from medical professionals.^17^ Large language models are an interesting perspective for free-text information retrieval.^18^ Recent advances in NLP, particularly models based on deep learning such as bidirectional encoder representations from transformers (BERT), have shown high accuracy in name entity recognition tasks in extracting clinical entities, outperforming traditional rule-based and machine learning models.^19^ Compared with rule-based systems like ours, these models automatically learn contextual dependencies, making them robust against variations in language structure and terminology. However, they also require substantial carbon, computational resources and large amounts of annotated training data, which limits their application in rare disease contexts. Domain-specific tuning can help address the issue of generalizability. For example, Zhou et al.^20^ developed a cancer domain-specific BERT model (CancerBERT), which significantly outperformed general BERT models by incorporating specialized vocabularies, achieving high F1 scores for cancer phenotyping. Such approaches could offer valuable insights into improving free-text extraction in rare cancers, including rare HNCs.

As seen in our study, combining multiple data sources (ICD-10 codes, ADICAP codes and free-text data) led to better algorithm performance, with PPV improving to 83% when at least two sources were used for identification. This approach reduced the reliance on a single, potentially error-prone source of information. We could hypothesize that including other data sources in our algorithm could also improve its performance. Another potential free-text data source is the MDM report. In our study, it was used only during the iteration phase of the creation of the NLP algorithm.

On the other hand, evaluating the performance of an algorithm designed to identify rare diseases, such as rare HNCs, in a large population poses several inherent challenges. The rarity of the condition—accounting for <5 in 10 000 individuals—makes it difficult to comprehensively assess algorithm performance due to class imbalance. That is why we chose to evaluate the algorithm over a preselected population (HNC patient cohort), a choice that could be debated.

The reliance on expert annotation for validation presents an additional challenge. In our study, only 100 cases were manually annotated by an HNC medical expert, limiting the ability to fully assess the algorithm’s generalizability. To enhance robustness, cross-validation techniques or external validation using data from other institutions could be employed. Although our study leveraged a large CDW from a single institution (AP-HP), testing the algorithm across different health care systems would help determine its adaptability to varying coding practices and pathology reporting formats. While the use of multimodal data sources increases accuracy, it also introduces complexity in terms of data processing. The extraction of free-text variables requires sophisticated NLP tools, which may not always generalize well to different institutions or health care systems. Additionally, the use of ADICAP codes and language-based tools can further restrict the generalizability of this method in other health care environments. Interoperability using mapping from ADICAP codes to Standard NOmenclature of MEDecine (SNOMED) standard concepts, for example, could be an option to explore.^21^

### Conclusions

Our study underscores the feasibility and advantages of using a multimodal approach to EHR data, combining structured codes (ICD-10, ADICAP) and free-text analysis through NLP, to identify patients with rare HNCs. Combining at least two data sources to create the consolidated rare HNC patient cohort offers significant improvements in accuracy over reliance on a single data source, an approach yielding a higher PPV and Sp. Future research should focus on expanding the validation of this approach in diverse health care environments, while also exploring advances in NLP. Analysis of a larger population could also help target which specific patient subcohort could benefit more from a multimodal or a unimodal approach, especially to know in which subcohort NLP algorithms perform best. Ultimately, leveraging large-scale EHR data could contribute to better understanding and management of rare cancers, but continued investment in data quality and processing technique is essential.
