# Supplementary figures and images for: Multimodal identification of a rare head and neck cancer patient cohort in the clinical data warehouse of Greater Paris Teaching Hospital
Source: ESMO Real World Data Digit Oncol. 2025 May 29;8:100151. doi: 10.1016/j.esmorw.2025.100151 (PMC12836562; doi:10.1016/j.esmorw.2025.100151)

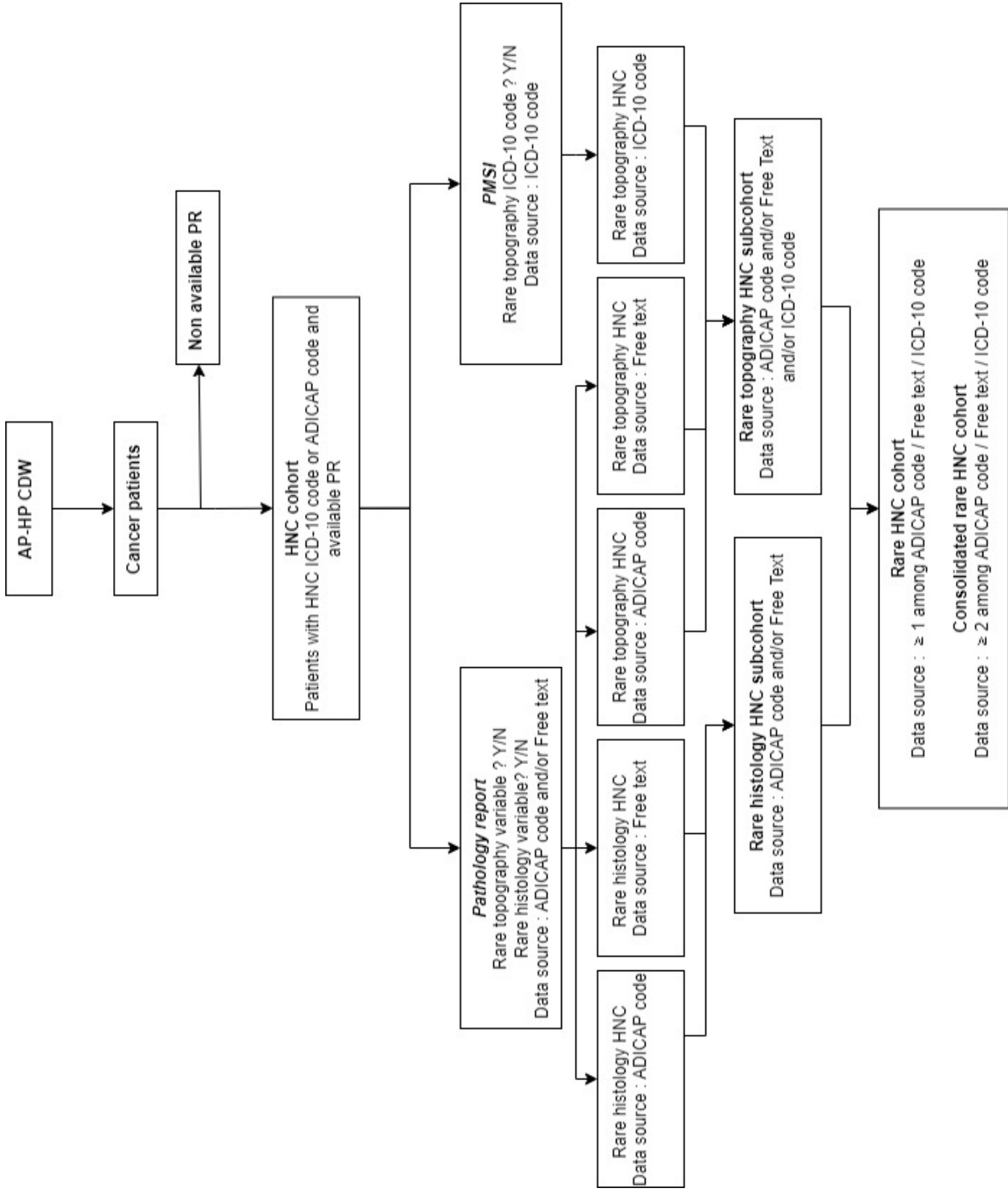

Supplement: Supplementary Fig 2 [file mmc2.pdf]

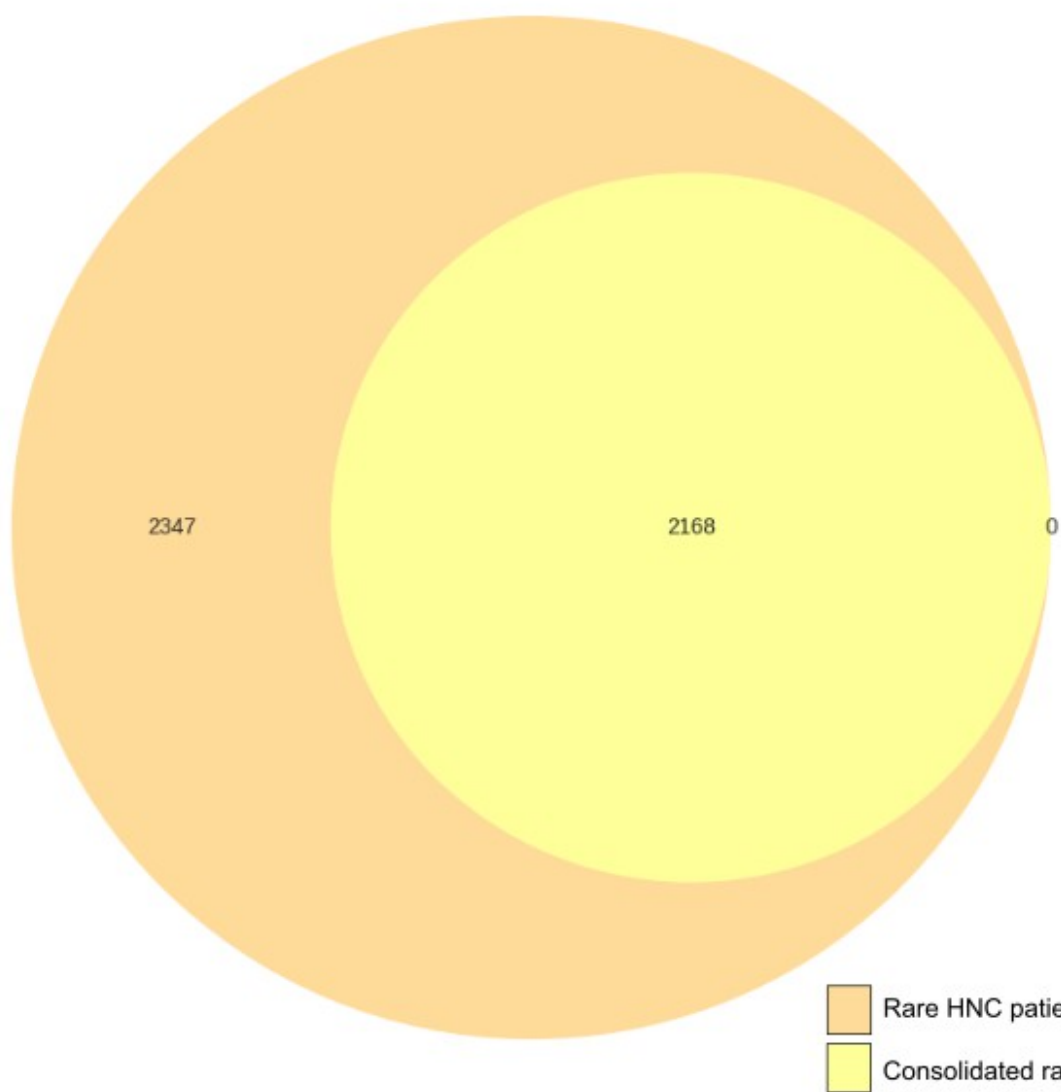

Supplement: Supplementary Fig 3 [file mmc3.pdf]
