# Supplementary material for: Multimodal identification of a rare head and neck cancer patient cohort in the clinical data warehouse of Greater Paris Teaching Hospital
Source: ESMO Real World Data Digit Oncol. 2025 May 29;8:100151. doi: 10.1016/j.esmorw.2025.100151 (PMC12836562; doi:10.1016/j.esmorw.2025.100151)
Supplement: Supplementary Data [file mmc4.docx]

**SUPPLEMENTARY MATERIAL**

**Supplementary Figure 1. RECORD Statement**

**Supplementary Figure 2. Summary of the methodology**

*Abbreviations: ADICAP: Association pour le Développement de l'Informatique en Cytologie et en Anatomie Pathologique, Association for the Development of Computer Science in Cytology and Pathological Anatomy. AP-HP CDW: Greater Paris Teaching Hospital (Assistance Publique – Hôpitaux de Paris, AP-HP) clinical data warehouse. HNC: head and neck cancer. ICD-10: International Classification of Diseases, 10th revision. NLP: natural language processing. PMSI : Programme de Médicalisation des Systèmes d'Information (nation claims data base).*

**Supplementary Figure 3. Venn diagram of the rare HNC cohort and the consolidated rare HNC cohort.**

*Abbreviation: HNC: head and neck cancer.*

**Supplementary Table 1. Cancer related ICD-10 codes.**

| Type of cancer by location | ICD-10 code |
| --- | --- |
| Anus | C21 |
| Biliary duct | C23 C24 D01.5 D37.6 |
| Bladder | C66 C67 C68 D09.0 D09.1 D41.2 D41.3 D41.4 D41.7 D41.9 |
| Bowel | C17 D01.4 D37.2 |
| Breast | C50 D05 D48.6 |
| Central Nervous System | C71 C72.0 C72.2 C72.3 C72.8 C72.9 D42 D43.0 D43.1 D43.2 D43.4 D43.7 D43.9 |
| Colon | C18 C19 D01.0 D01.1 D37.3 D37.4 |
| CUP | C76 C80 C97 D09.7 D09.9 D48.7 D48.9 D48.3 |
| Endometrium | C54 C55 D07.0 D39.0 |
| Eye | C69 D09.2 |
| Gastric | C16 D00.2 D37.1 |
| Head and neck | C0 C10 C11 C12 C13 C14 C30 C31 C32 D00.0 D02.0 D37.0 D38.0 |
| Kidney | C6.4 C6.5 D41.0 D41.1 |
| Leukemias | C91 C92 C93 C94.0 C94.1 C94.2 C94.3 C94.4 C94.5 C94.7 C95 |
| Liver | C22 |
| Lung | C33 C34 D02.1 D02.2 D38.1 |
| Lymphomas | C81 C82 C83 C84 C85 C86 |
| Melanoma | C43 D03 |
| Mesothelioma | C45.0 C45.1 C45.2 C45.7 C45.9 |
| Myeloma | C90 |

**Supplementary Table 2. HNC related ICD-10 codes and ADICAP codes.**

| ADICAP codes regular expressions | [A-X][A-Z]A[ZACFHLOPS][ABCDEFGL-X][7-9EM][A-Z][0-9]  [A-X][A-Z]B[ZADGLMPSVX][ABCDEFGL-X][7-9EM][A-Z][0-9]  [A-X][A-Z]O[RL][ABCDEFGL-X][7-9EM][A-Z][0-9]  [A-X][A-Z]XF[ABCDEFGL-X][7-9EM][A-Z][0-9] |
| --- | --- |
| ICD-10 codes | 'C00','C000','C001','C002','C003','C004','COO5','C006','C007','C008',  'C009','C01','C02','C020','C021','C022','C023','C024','C028','C029','C0210', 'C03','C030','C031','C039','C04','C040','C041','C048','C049','C05','C050', 'C052','C058','C059',’C06','C060','C061','C062','C068','C069','C09','C090', 'C091','C098','C099','C10','C100','C101','C102','C103','C104','C108','C109', 'C12','C13','C130','C131','C132','C138','C139','C14','C140','C141','C142',  'C148','C32' |

*Abbreviations : ADICAP: Association pour le Développement de l'Informatique en Cytologie et en Anatomie Pathologique, Association for the Development of Computer Science in Cytology and Pathological Anatomy. HNC: head and neck cancer. ICD-10: International Classification of Diseases, 10th revision.*

**Supplementary Table 3. Regular expressions from pathology reports ADICAP codes depending on the variable.**

| Variable | ADICAP code regular expressions |
| --- | --- |
| Rare topography HNC | [A-X][A-Z]A[CFS][AC-X][6-9EM][A-Z][0-9]  [A-X][A-Z]A[CFS]B7[FGKLN][0-9]  [A-X][A-Z]OR[AC-X][6-9EM][A-Z][0-9]  [A-X][A-Z]ORB7[FGKLN][0-9]  [A-X][A-Z]B[PMS][AC-X][6-9EM][A-Z][0-9]  [A-X][A-Z]B[PMS]B7[FGKLN][0-9]  [A-X][A-Z]XF[ACDFGLMNPQRSTVX][6-9EM][FGKLN][0-9]  [A-X][A-Z]OL[AC-X][6-9EM][A-Z][0-9]  [A-X][A-Z]OLB7[FGKLN][0-9] |
| Rare histology HNC | [A-X][A-Z]A[ZACFHLOPS][ACDFGLMNP-X][7-9EM][A-Z][0-9]  [A-X][A-Z]B[ZADGLMPSVX][ACDFGLMNP-X][7-9EM][A-Z][0-9]  [A-X][A-Z]O[LR][ACDFGLMNP-X][7-9EM][A-Z][0-9]  [A-X][A-Z]XF[ACDFGLMNP-X][7-9EM][A-Z][0-9]  [A-X][A-Z]A[ZACFHLOPS]B[7-9EM][FKGLN][0-9]  [A-X][A-Z]B[ZADGLMPSVX]B[7-9EM][FKGLN][0-9]  [A-X][A-Z]O[LR]B[7-9EM][FKGLN][0-9]  [A-X][A-Z]XFB[7-9EM][FKGLN][0-9] |

**Supplementary Table 4. Regular expressions from pathology reports free text depending on the variable.**

| Variable | Regular expressions |
| --- | --- |
| Rare topography  HNC | " nasal", 'cornet', 'orifices? narin', 'vestibule?s? narin', 'turbin', 'olfactif?v?e?s?', 'cloison', 'septum', 'septal', 'vomer', 'nasopharyn', 'cavum', 'rhinopharyn'  'petrectomie', 'mastoid', 'oreille moyenne', '\\brocher',                                      'sac endo[- ]?l[iy]mph?atique','meat auditif', 'conduit auditif externe','\\bcae\\b', 'tympan', 'marteau',"naso[- ]?sinusien", "maxill[aoe]", "ethmoid", "sinus frontal", "sphenoid","sino-?nasal", "cloison inter-?sinuso-?nasale", "parotid", "sous[ -]?max", "sous[ -]?mandibul", "sub[ -]?mandibul", "sub[ -]?ling", "glandes? salivaires? accessoires?",'massif faciale?','oss?e?u?s?e? du cr[aâ]ne','z[iy]goma', 'vo[ûu]te cr[âa]n','os frontal','primiti.{1,15}mandibul','primiti.{1,15}maxill', 'primiti.{1,15}temporal','infra[- ]?temporale?','pt[ée]r[yi]go','lacr[yi]mal', 'odontog[eé]n','oss?e?e?u?s?e de laface','oss?e?e?u?x? de la face','oss?e?u?x du cr[aâ]ne','os pari[ée]tal','os temporal','clivus','clival','odonto[iï]','foramen jugulaire' |
| Rare histology  HNC | '[ée]pend[iy]mome','ad[eé]no[- ]?carcinome','ad[eé]noK','am[eé]lo[- ]?blast','angio[- ]?m[iy]xomeagressif', 'astroc[yi]tome', 'c[iy]stad[eé]no[ -]?carcinome','carcinome [aà] cel?lules claires','carcinome [àa]grandes cel?lules','carcinome [àa] petites cel?lules ','carcinome anaplasique', 'carcinome embryonn?aire','carcinome intra[- ]?osseux','carcinome muco[- ]?ée]pidermo[ïi]de', 'carcinome neuro[- ]?endocrine', 'chordome','chondro[- ]?blastome','cylindrome','d[yi]s[- ]?germinome','Dar?rier[ -]?Fer?rand','fibro[ -]?xanth?ome malin','fibro[- ]?blastome', 'ganglio[- ]?gliome','gl[iy]o[- ]?blastome','h[ée]mang[iy]o[- ]?endoth?[ée]liome','h[ée]mang[iy]o[- ]?p[ée]r[iy][- ]?c[yi]tome malin',                'h[iy]st[iy]o[- ]?c[yi]to[- ]?xanth?ome malin','h[iy]st[yi]o[ -]?cyto[ -]?fibrome angiomato[ïi]de','h[iy]st[yi]o[- ]?c[iy]tome fibreux malin','h[iy]st[yi]oc[yi]tose malig?ne','m[ée]lanome','m[ée]ningiome anaplasique','m[ée]ningiome malin','m[ée]sench[yi]ome malin','m[ée]soth?[ée]l[iy]ome','m[yi][ée]lome','neuro[- ]?blastome','neuro[- ]?fibrome anaplasique','neuro[- ]?fibrome malin','ol[iy]go[- ]?dendro[ -]?gl[iy]ome','para[- ]?gangl[iy]ome malin','ph[ée]o[- ]?ch?romo[- ]?c[yi]tome malin','pol[iy]-embr[yi]ome','s[ée]minome','sarcom','sc?hwann?ome malin','t[ée]rato[- ]?carcinome','t[eé]ratome (mature )?canc[eé]risé','t[ée]ratome imm?ature?','t[eé]ratome transformé','tumeur [aà] cellules? claires?','tumeur acineuse','tumeur ad[eé]nomateuse','tumeur carc[iï]no[iï]de','tumeur dys[- ]?g[eé]n[eé]tique canc[ée]ris[ée]e?','tumeur dys[- ]?g[eé]n[eé]tique malig?ne','tumeur fibreuse','tumeur fibro[- ]?blastique','tumeur fibro[- ]?hist[yi]o[- ]?c[iy]tique','tumeur germinale?','tumeur germinative','tumeur m[ée]lanique','tumeur muco[- ]?[eé]pidermo[iï]de','tumeur odontog[eè]ne malig?ne','UCNT','xanth?ome malin','[ée]sth?[ée]sio[- ]?neuro','carcinome indif?f[eé]renci', 'carcinome ver?ruqueux', 'carcinome cuniculatum', 'carcinome .{0,30}basalo[iï]de', 'carcinome .{0,30}papillaire', ‘carcinome .{0,30}acanth?ol[yi]t[yi]que', 'carcinome ad[ée]no[iï]de k[yi]stique', 'CAK', 'carcinome .{0,30}fusiforme','carcinome .{0,30}ad[ée]no[ -]?squameux', 'carcinome canalaire salivaire' |
| Common topography  HNC | 'buc?cal', '(?<!sub-)lingual', '(?<!sub)lingual','gingiv','palais','\\bmandibul','face interne (de )?joue','trigone r[ée]tro[ -]?molair','[\\brbmi\\b](file:///brbmi/b)','amygdal','\\btrm\\b','voile','v[ée]laire','langue','laryn', 'pharyn', 'glotti','face interne de l[èe]vre','sinus p[yi]r[yi]forme','[ée]piglott','bande ventriculair','corde vocal', 'vall?[eé]cul','pli vocal','carrefour des trois replis','carrefour des 3 replis','r[ée]gion des trois replis','r[ée]gion des 3 replis','aryth?[ée]no[ïi]de', 'plancher buccal','cavit[eé] buccal',"cavit[eé] oral",'glosso' |
| Common histology  HNC | '\\bcarcinome\\b', 'tumorale?','cancer ','n[ée]oplasie','n[ée]oplasme malin' |

**Supplementary Table 5. Performance metrics of the algorithm for identifying rare HNC patient subcohorts depending on data sources.**

| **Rare topography HNC subcohort** |  | **TP** | **FP** | **FN** | **TN** | **Total** |  | **Sensitivity** | **Specificity** | **PPV** | **NPV** |
| --- | --- | --- | --- | --- | --- | --- | --- | --- | --- | --- | --- |
| ICD-10 code |  | 14 | 10 | 4 | 72 | 100 |  | 78% | 87% | 58% | 95% |
| ADICAP code |  | 11 | 6 | 7 | 76 | 100 |  | 61% | 93% | 65% | 92% |
| NLP processed free text |  | 17 | 13 | 1 | 69 | 100 |  | 94% | 84% | 57% | 99% |

| **Rare histology HNC subcohort** |  | **TP** | **FP** | **FN** | **TN** | **Total** |  | **Sensitivity** | **Specificity** | **PPV** | **NPV** |
| --- | --- | --- | --- | --- | --- | --- | --- | --- | --- | --- | --- |
| ADICAP code |  | 13 | 4 | 5 | 78 | 100 |  | 72% | 95% | 76% | 94% |
| NLP processed free text |  | 17 | 13 | 1 | 69 | 100 |  | 94% | 84% | 57% | 99% |

*Abbreviations : ADICAP: Association pour le Développement de l'Informatique en Cytologie et en Anatomie Pathologique, Association for the Development of Computer Science in Cytology and Pathological Anatomy. HNC: head and neck cancer. ICD-10: International Classification of Diseases, 10th revision. FN: false negatives. FP: false positives. NLP: Natural Language Processing. NPV: negative predictive value. PPV: Positive predictive value. TN: true negative. TP: true positives.*
